# Finite element modeling of meniscal tears using continuum damage mechanics and digital image correlation

**DOI:** 10.1038/s41598-023-29111-z

**Published:** 2023-03-10

**Authors:** Derek Q. Nesbitt, Dylan E. Burruel, Bradley S. Henderson, Trevor J. Lujan

**Affiliations:** 1 Biomedical Engineering Doctoral Program, Boise State University, Boise, ID USA; 2 Department of Mechanical and Biomedical Engineering, Boise State University, 1910 University Drive, Boise, ID 83725-2085 USA

**Keywords:** Biomedical engineering, Mechanical engineering, Computational science, Musculoskeletal abnormalities

## Abstract

Meniscal tears are a common, painful, and debilitating knee injury with limited treatment options. Computational models that predict meniscal tears may help advance injury prevention and repair, but first these models must be validated using experimental data. Here we simulated meniscal tears with finite element analysis using continuum damage mechanics (CDM) in a transversely isotropic hyperelastic material. Finite element models were built to recreate the coupon geometry and loading conditions of forty uniaxial tensile experiments of human meniscus that were pulled to failure either parallel or perpendicular to the preferred fiber orientation. Two damage criteria were evaluated for all experiments: von Mises stress and maximum normal Lagrange strain. After we successfully fit all models to experimental force–displacement curves (grip-to-grip), we compared model predicted strains in the tear region at ultimate tensile strength to the strains measured experimentally with digital image correlation (DIC). In general, the damage models underpredicted the strains measured in the tear region, but models using von Mises stress damage criterion had better overall predictions and more accurately simulated experimental tear patterns. For the first time, this study has used DIC to expose strengths and weaknesses of using CDM to model failure behavior in soft fibrous tissue.

## Introduction

Meniscal tears are one of the most common musculoskeletal injuries, with nearly a half-million meniscal tear injuries occurring in the U.S. each year^1^. Once torn, the load attenuating capability of the semi-circular meniscus can become permanently compromised, leading to chronic knee joint pain and instability^2^. The meniscus also has a diminishing capacity to heal with age^3^ and surgical interventions that remove damaged meniscal tissue increase the likelihood of osteoarthritis^4,5^. With the lack of effective treatment options to fully restore meniscus function, the prevention of meniscal tear injuries is of utmost importance. Meniscal tears are classified by their orientation relative to the anisotropic collagen type I fiber matrix^6,7^, which is primarily aligned circumferentially to resist the large tensile or hoop stresses that develop during joint compression^8^. Tears can occur alongside the fibers through the ground substance (e.g. horizontal and vertical tears), or can disrupt or break the circumferential fibers (e.g. radial and flap tears). Despite the prevalence and impact of this injury, the physical mechanism of meniscal tears is poorly understood.

Computational tools like finite element (FE) analysis can be utilized to help understand injury mechanisms in soft tissue, as well as inform the development of injury prevention strategies^9,10^. While many three-dimensional FE models of soft tissue structures in the knee and other joints have been created to analyze stresses and strains during normal and pathological activities^11–13^, FE models have not been previously developed to simulate meniscal tears. One way that FE can be effectively used to simulate meniscal tears is by using Continuum Damage Mechanics (CDM) to model material weakening and eventual loss of load bearing capacity due to the onset and propagation of damage. Material damage can be described as the breaking of chemical bonds^14^ or coalescence of microscopic voids^15^. This damage will irreversibly reduce the material’s ability to resist deformation until material separation occurs (tissue tearing). Several studies have used CDM to successfully simulate the stress-softening behavior observed in stress–strain curves of soft tissue under tension^16–20^, but no previous soft tissue study has used FE models to analyze the localized failure behavior in the tear region predicted by CDM.

A critical step in computational research is to perform a validation study to determine whether the model is able to simulate independent experimental data not used to calibrate (fit) the model parameters^21^. A validation technique useful for modeling mechanical failure is to compare FE predicted surface strains in the tear region to full field strain maps experimentally measured using digital image correlation (DIC)^22–28^. This approach is advantageous as it allows for the calibration and validation of a computational model using the same experimental data set; first by tuning model parameters to the grip-to-grip stress–strain behavior (or force–displacement behavior), then validating the model by comparing the localized surface strains predicted by the model and measured experimentally. This validation method has been used in FE models of bone fracture ^22–24^, but has not previously been applied to soft tissue. However, a study by Von Forell and Bowden did make a qualitative comparison between FE and DIC shear strains in tendon, demonstrating that CDM has potential to predict observed deformations in the tear region when using appropriate formulations to evolve damage^29^. Similarly, findings from our previous experimental work that used DIC with high-speed video to characterize tensile failures in human meniscus^30^ can inform a constitutive framework for a physiological damage model. We found that when loaded along the fiber network (longitudinal), failures followed the plane of maximum shear strain. This suggests that macroscale fiber failures are driven by distortion energy, and therefore von Mises stress may be an appropriate damage criterion^30,31^. When loaded normal to the fibers (transverse), failures occurred along the plane of maximum normal strain. This suggests that macroscale ground substance failures are driven by first principal strain^30^, and maximum normal strain may be an appropriate damage criterion. Further, our previous DIC measurements of localized strains in the tear region at precise points on the stress–strain curve can be used to validate whether CDM is capable of recreating the physiological strains occurring during meniscal tears.

The objective of this work was to determine if CDM can predict the anisotropic, macroscale failure behavior of human meniscal tissue under tensile loading. We hypothesize that a transversely isotropic hyperelastic damage model using von Mises stress damage criterion for fiber failures, and maximum normal strain damage criterion for ground substance failures, will be able to reproduce the grip-to-grip force–displacement behavior of quasi-static tensile tests and planar strain in the tear region.

## Methods

### Overview

Finite element analysis was used with CDM to simulate uniaxial tensile experiments of human meniscus. We analyzed two loading configurations (longitudinal, transverse) and two CDM damage criterion (von Mises stress, maximum normal strain). Model parameters were tuned to experimental force–displacment curves and quality-of-fit was measured. The tissue strains within the tear region of interest (ROI) were then compared to those measured experimentally by DIC in our previous work^30^ to determine the accuracy of the model in predicting meniscal tears.

### Tensile tests using DIC

An in-depth description of the experimental methods for the uniaxial tensile tests can be found in our previous experimental paper^30^. In this prior study, human menisci were harvested from cadaveric knees that were obtained through an accredited tissue bank (Science Care Inc., Pheonix, AZ), and all experimental protocols were approved by the Institutional Biosafety Committee at Boise State University. In brief, 40 monotonic uniaxial tensile tests (loading rate = 1% strain/s) were conducting using specimens from five young human cadaveric menisci (age = 33 ± 5 years; BMI = 21 ± 1) and five older human cadaveric menisci (age = 72 ± 7 years; BMI = 26 ± 5). All knees had no medical history of injury or visible signs of damage or degeneration. Thin layers of meniscus were cut from both the anterior and posterior region^32^, and punched into dumbbell shaped coupons^33^ either along the preferred fiber direction (longitudinal) or normal to the preferred fiber direction (transverse). Specimens were speckled for DIC analysis, preloaded, mechanically preconditioned, and then preloaded again prior to being pulled to failure in tension. A high-speed camera and DIC software were used to measure planar strains from the start of testing to tissue separation (failure). Planar tissue strains were calculated in an ROI that was centered along the tear line of action and spanned the width and thickness of the coupon with a vertical height of 2 mm. These ROI strains were calculated at ultimate tensile stress, where the tissue begins losing load bearing capacity. Axial force and grip displacements were used to calculate the overall stress–strain curve for each specimen.

### Damage model

The selected constitutive model was a transversely isotropic hyperelastic^34^ damage model available in FEBio^35^, where the material’s strain energy density *Ψ* is uncoupled into hydrostatic and deviatoric components of the ground substance *F*_*1*_ and fiber matrix *F*_*2:*_

1 $$\Psi ={F}_{1}\left({\widetilde{I}}_{1}, {\widetilde{I}}_{2}\right)+{F}_{2}\left(\widetilde{\lambda }\right)+\frac{K}{2}{\left(\mathit{ln}\left(J\right)\right)}^{2}.$$

Here *Ĩ*_*1*_ and* Ĩ*_*2*_ are the first and second invariants of the deviatoric portion of the right Cauchy-Green deformation tensor, *λ̃* is the deviatoric part of stretch along the fiber direction, *J* is the volume change ratio, and *K* is bulk modulus, which was set to 1000 to enforce near-incompressibility^36^. Following previous research in ligament modeling^37,38^, we used a Veronda–Westmann formulation for the ground substance (Eq. 2), where strain energy is dependent on two material coefficients (*C*_1_, *C*_2_). A piecewise exponential-linear function was used for the fiber network (Eq. 3), where the transition fiber stretch *λ*_*m*_ defines the transition between the toe and linear region.

2 $${F}_{1}={C}_{1}\left({e}^{\left({C}_{2}\left({\widetilde{I}}_{1}-3\right)\right)}-1\right)-\frac{{C}_{1}{C}_{2}}{2}\left({\widetilde{I}}_{2}-3\right),$$

3 $$\widetilde{\lambda }\frac{\partial {F}_{2}}{\partial \widetilde{\lambda }}=\left\{\begin{array}{ll}0& \widetilde{\lambda }\le 1 \, \\ {C}_{3}\left({e}^{{C}_{4}\left(\widetilde{\lambda }-1\right)}-1\right) \, & 1<\widetilde{\lambda }<{\lambda }_{m}\\ {C}_{5}\widetilde{\lambda }+{C}_{6} \, & \widetilde{\lambda }\ge {\lambda }_{m.}\end{array}\right.$$

When the fiber stretch is below *λ*_*m*_, the function is dependent on two material coefficients that effectively control fiber straightening (*C*_3_, *C*_4_). At a fiber stretch above *λ*_*m*_, the fibers take on an approximately linear character, and are dependent on fiber modulus *C*_5_, where *C*_6_ ensures that stress is continuous at the transition stretch. The calculated strain energy density from the hyperelastic model is converted to an effective undamaged Cauchy stress ${{\varvec{\sigma}}}_{o}$.

In order to model stress softening behavior, a quintic polynomial cumulative distribution function was implemented to apply damage evolution.

4 $$D\left(\Xi \right)=\left\{ \begin{array}{ll}0& \Xi \le {\mu }_{min} \\ {x}^{3}\left(6{x}^{2}-15x+10\right) & { \mu }_{min}<\Xi <{\mu }_{max} , x=\frac{{\Xi -\mu }_{min}}{{\mu }_{max}-{\mu }_{min}} \\ {D}_{max}& {\Xi \ge \mu }_{max}\end{array}\right..$$

Here *D* ranges from zero to a maximum damage (*D*_*max*_), controlled by the limits *μ*_*min*_ and *μ*_*max*_. Damage *D* is a function of the selected damage criterion (*Ξ*): von Mises stress (Eq. 5) or maximum normal Lagrange strain (Eq. 6).

5 $$\Xi =\sqrt{\frac{3}{2}{\widetilde{{\varvec{\upsigma}}}}_{o}{:\widetilde{{\varvec{\upsigma}}}}_{o}},$$

6 $$\Xi =Max \left({E}_{1},{E}_{2},{E}_{3}\right),$$

where ${\widetilde{{\varvec{\upsigma}}}}_{o}$ is the deviatoric part of undamaged stress ${{\varvec{\upsigma}}}_{o}$, and *E*_1_, *E*_2_, and *E*_3_ are the principal values of Lagrange strain **E**. This damage was applied to both the fiber and ground substance terms (Eqs. 2–3). Finally, Cauchy stress **σ** was calculated by scaling the effective undamaged stress tensor **σ**_**o**_ with scalar damage *D*.

7 $${\varvec{\sigma}}=\left(1-D\right){{\varvec{\sigma}}}_{0}.$$

### Finite element mesh and coupon geometry

**Figure 1:**
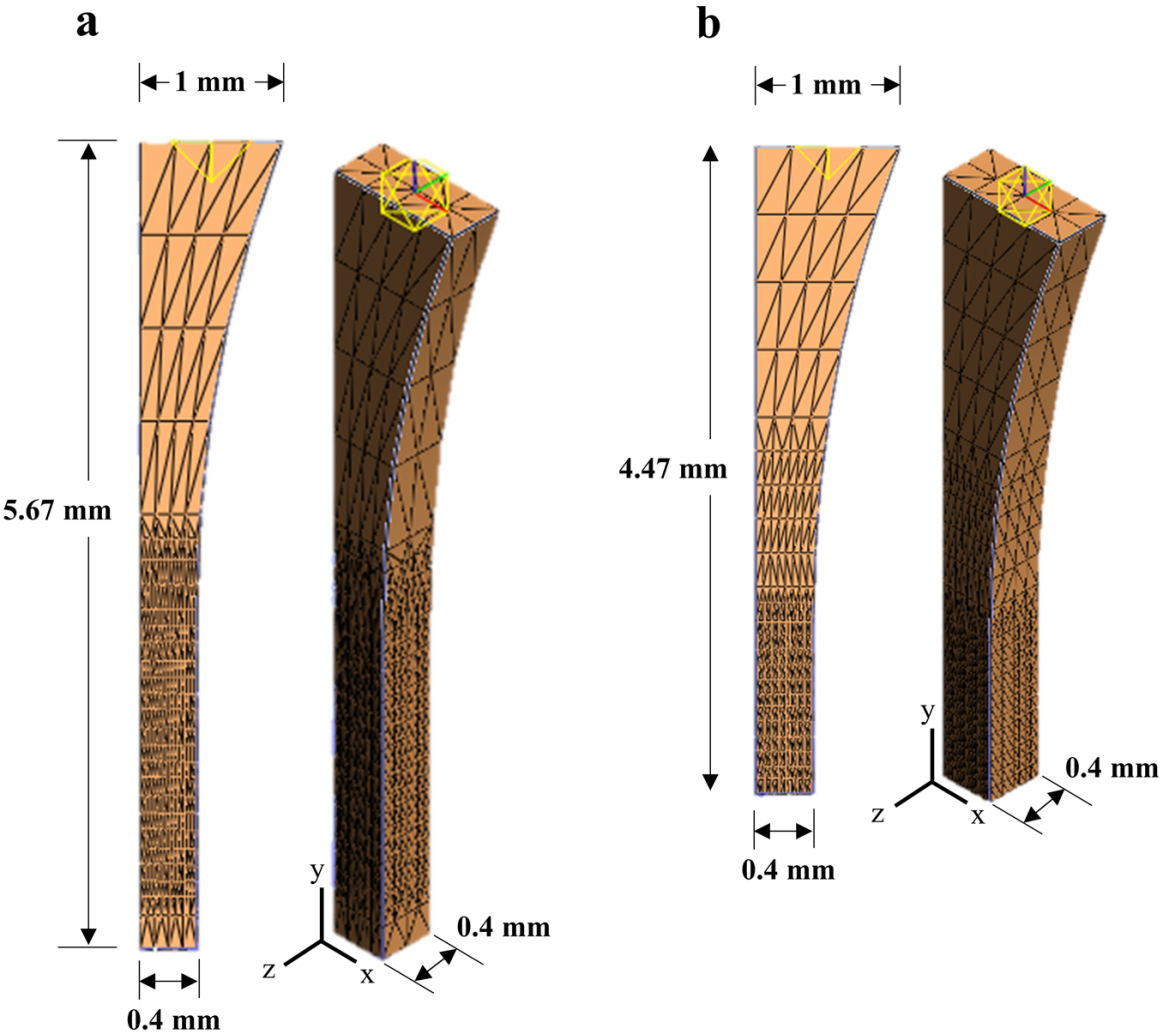
Coupon dimensions and mesh for all (**a**) longitudinal and (**b**) transverse models.

**Figure 2:**
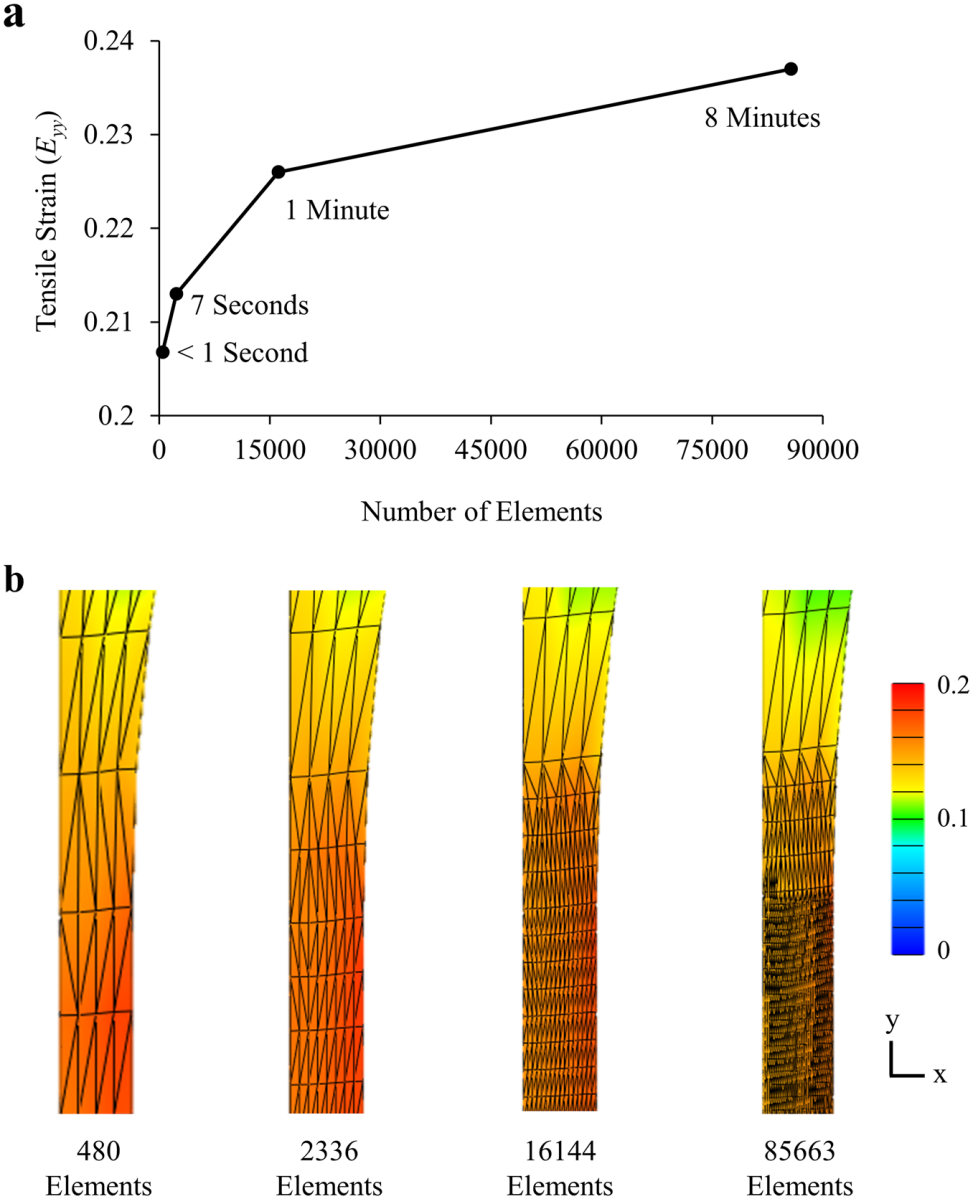
Mesh convergence study. (**a**) Increasing mesh size resulted in longer run times and greater tensile strain (*E*_yy_) inside the tear region that gradually plateaued. (**b**) Strain maps (*E*_*yy*_) of different mesh sizes. This study used 16,144 elements.

Computational effort was reduced by modeling 1/8th of dumbbell shaped coupons along three planes of symmetry (Fig. 1). Model dimensions reflected the average coupon dimensions measured experimentally for longitudinal (Fig. 1a) and transverse (Fig. 1b) specimens^30^. For boundary conditions, the grip was modeled as a rigid body with an irrotational sliding elastic tension contact at the top of the coupon, while the axis normal to each plane of symmetry was fixed. The three-dimensional models were meshed with 10 × 8 × 2 linear tetrahedral elements, then refined by four-fold in an approximately 2.5 mm tall region spanning the width and thickness of the coupon where the highest strain concentrations occurred prior to failure. This localized refinement was done to help reduce the premature model termination we observed in coarse meshes due to damage localization. A mesh convergence study was conducted to determine the effect of mesh refinement on tensile strain along the loading axis (*﻿E*_*yy*_) when a grip-to-grip stretch of 1.20 was applied. Tensile strain was selected as the criterion for mesh convergence since strain is the outcome measure we’re comparing to our prior experimental results^30^. The mesh convergence study found that refinement increased the tensile strain (*E*_*yy*_; averaged in the ROI), although this increase exhibited a plateauing trend (Fig. 2a). For this study, we selected a mesh size (16,144 elements; Fig. 2b) that was computationally efficient (~ 1 min runtime) and gave strain results near the projected curve plateau (Fig. 2a). Linear tetrahedral elements were selected for ease of mesh generation compared to more complex elements^39^.

### Parameter optimization

Tensile tests were simulated by displacing the rigid body grip by the specimen specific grip-to-grip stretch, and optimizing the model parameters to make the axial forces occurring at the rigid body grip be one-quarter of the experimental axial forces measured at the grip. The reason the experimental axial force was reduced by one-quarter was to account for the model coupons having one-quarter of the average cross-sectional area of the experimental coupons (Fig. 1). Force–displacement curves were converted to stress–strain curves using the initial length and initial cross-sectional area of the model coupon. In total, there were nine parameters to fit in the hyperelastic damage model: two ground substance parameters, four fiber parameters, and three damage parameters. The two parameters of the elastic ground substance (Eq. 3) were first fit to stress–strain curves of transverse specimens up to the yield point (point of maximum slope determined with Dots-on-Plots^40^) using the Levenberg–Marquardt parameter optimization module in FEBio^35^. The four parameters of the elastic fiber network were next fit to the stress–strain curves of longitudinal specimens up to the yield point, also using FEBio’s parameter optimization module. For these optimizations, initial guesses for *C*_1_,* C*_5_, and *λ*_*m*_ were based on our previously measured mechanical properties^30^, with optimization limits of more than ± two standard deviations. The initial guess for *C*_2_ was set to one to enforce near incompressibility, and the initial guesses for the remaining elastic parameters were similar to a prior study^37^ with limits of approximately double and one-half the initial guess, respectively. When optimization returned a parameter extremum, that extremum was expanded by 30% and optimization was reperformed. This process was repeated until the optimization returned parameters that were not extrema. The average values for the fitted elastic parameters were *C*_1_ = 0.78 ± 0.66 MPa, *C*_2_ = 1.20 ± 1.8, *C*_3_ = 0.43 ± 0.23 MPa, *C*_4_ = 40.83 ± 9.95, *C*_5_ = 119.63 ± 50.07 MPa, and *λ*_*m*_ = 1.048 ± 0.007.

**Figure 3:**
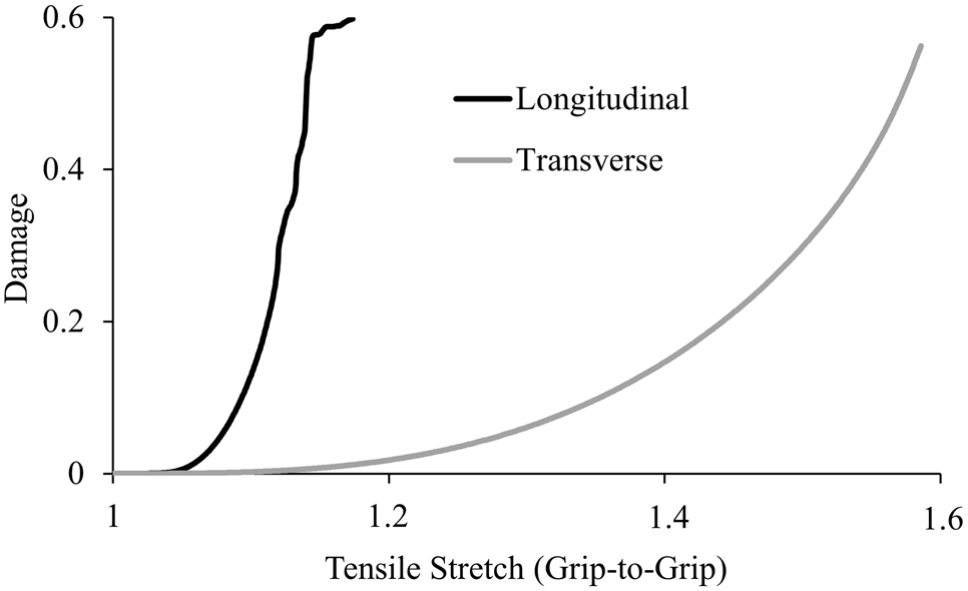
Maximum damage in the tear region of two representative specimens.

Once the elastic parameters were optimized, the three damage parameters were selected to best fit the stress–strain curve between the yield point and ultimate tensile strength (UTS). Automated optimization of damage parameters was not feasible, as the optimization module would often select a combination of parameters that resulted in early model termination and thereby caused the optimization routine to halt. Instead, model damage parameters were manually fit with specific success criteria, starting with initial guesses for *µ*_*min*_ and *µ*_*max*_ at yield strain and ultimate strain, respectfully. Models were considered successfully fit once model predicted ultimate stress and strain were within 0.2 MPa and 3% strain of the experimental ultimate stress and strain for longitudinal models, respectively; and 0.03 MPa and 3% strain for transverse models, respectively. A successful fit also required a post-UTS reduction in stress of at least 1% and 0.5% from the ultimate stress for the longitudinal and transverse models, respectively. The value of *D*_*max*_ was kept below 1 (no load carrying capacity when *D* = 1) to help avoid model convergence issues that have been previously described^29,41^. Different damage parameters were used for the longitudinal and transverse specimens (Table 1), as damage onset of the ground substance occurs at much greater strains then fiber damage (Fig. 3).

**Table 1﻿:** Damage parameters for models loaded longitudinal or transverse to the preferred fiber direction.

	Von Mises stress damage criterion			Max normal strain damage criterion		
	*µ*_*min*_	*µ*_*max*_	*D*_*max*_	*µ*_*min*_	*µ*_*max*_	*D*_*max*_
Longitudinal	8.83 ± 9.26	43.66 ± 30.17	0.58 ± 0.08	0.18 ± 0.21	0.48 ± 0.26	0.61 ± 0.09
Transverse	0.19 ± 0.26	2.20 ± 1.98	0.65 ± 0.13	0.12 ± 0.16	1.18 ± 0.68	0.73 ± 0.13

### Model evaluation and validation

**Figure 4:**
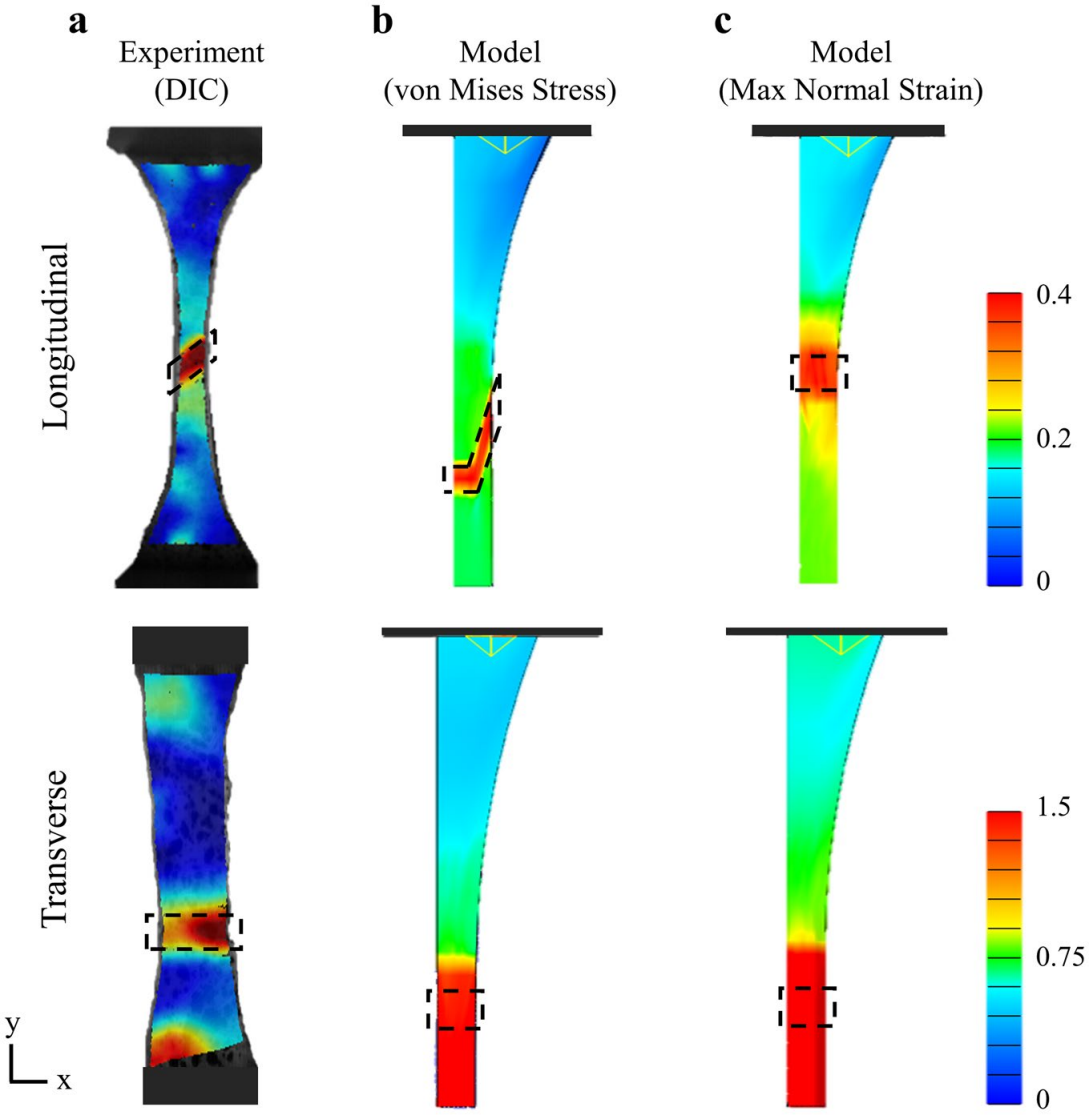
Normal Lagrange strains (*E*_yy_) from experiments and FE models for longitudinal and transverse specimens. (**a**) The experimental tear pattern and strains in the tear region (ROI) measured with DIC were compared to model predictions using (**b**) von Mises stress damage criterion, and (**c**) maximum normal strain damage criterion. ROI are shown by the dashed black box.

The failure ROI for the model spanned the vertical coupon edges and was determined as one element above and below the line-of-action of greatest damage concentration. This resulted in ROI height spans of 0.2 mm for all transverse models, 0.24 mm for longitudinal models with the von Mises damage criterion, and 0.3 mm for longitudinal models with the maximum normal strain damage criterion. These ROI heights are comparable to the 0.2 mm ROI heights from the DIC experiment^30^ (Fig. 4). The location and shape of this ROI was the same for all models within each group. Average normal and shear Lagrange strains (*E*_yy_, *E*_xx_, *E*_xy_), principal strains (*E*_1_, *E*_2_), and maximum shear strain (*γ*_*max*_), of the element surfaces in the ROI were output to a logfile, averaged, and compared to the planar Lagrange strains measured by DIC during the uniaxial tensile experiment (Fig. 4a)^30^.

### Statistics

Quality of fit between the experimental and model force–displacement curves was determined by calculating the NRMSE (normalized to mean stress) and coefficient of determination (*R*^2^). The effect of damage criteria on quality of fit was determined using a one-way ANOVA. Differences in ultimate tensile force (grip-to-grip), ultimate tensile stretch (grip-to-grip), and average Lagrange strains within the tear region at UTS between the models and experiment were determined with a repeated measures ANOVA. All significance in this study was set at *p* < 0.05.

## Results

### Model fit to force–displacement curves

**Figure 5:**
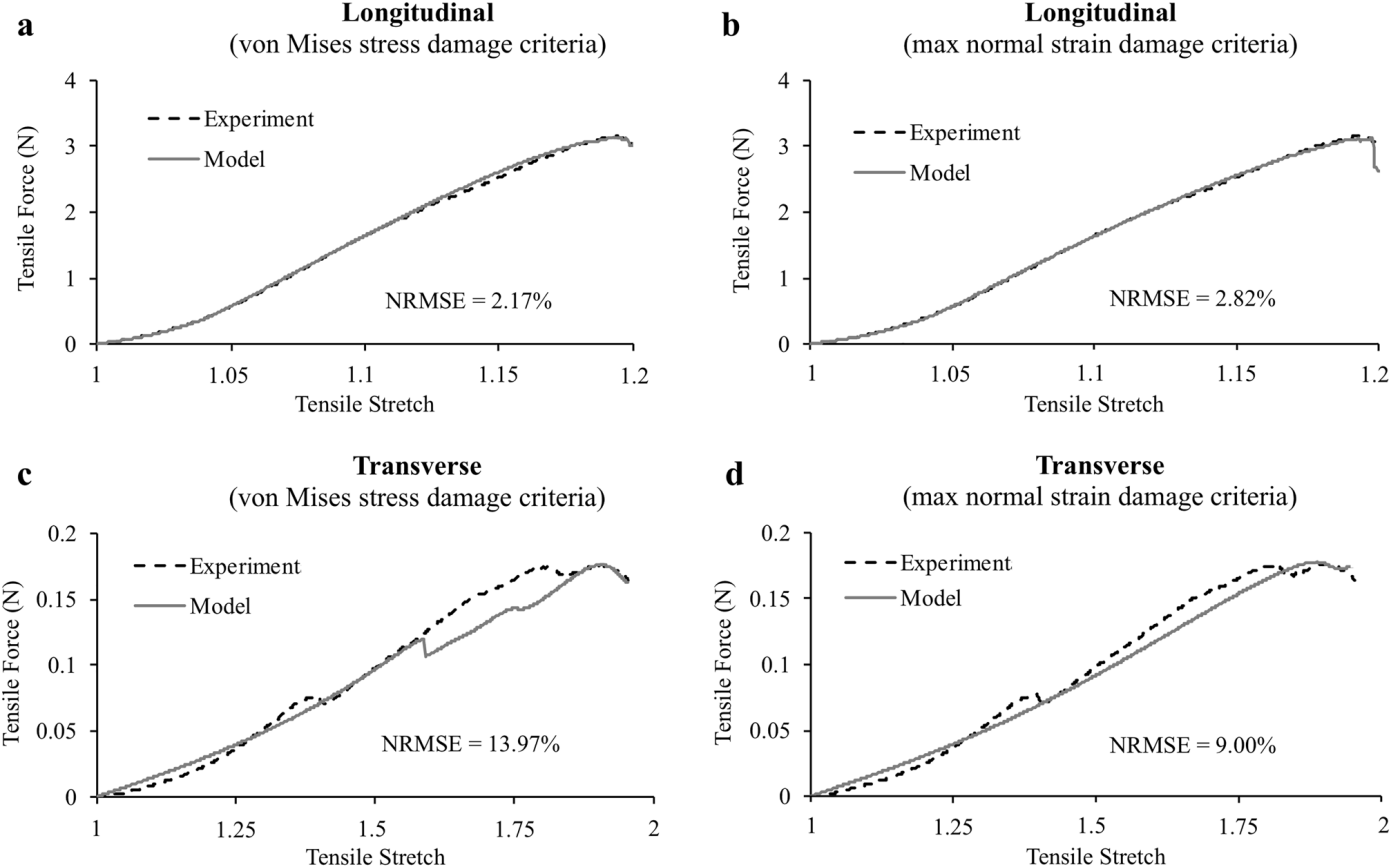
Representative model fits to experimental grip-to-grip force–displacement curves (we converted grip displacement to tensile stretch). (**a**) When modeling the fiber response (longitudinal), von Mises stress damage criterion had better fits compared to the (**b**) maximum normal strain damage criterion. (**c**) However, von Mises stress damage criterion had slightly poorer quality of fits compared to (**d**) maximum normal strain damage criterion when modeling the ground substance (transverse).

Finite element models were successfully fit to experimental grip-to-grip force–displacement curves (Fig. 5). Longitudinal models utilizing the von Mises stress damage criterion had significantly better quality of fits to experimental force–displacement curves relative to the maximum normal strain damage criterion (*p* = 0.04), with average NRMSEs of 2.10 ± 1.25% (*R*^2^ = 0.999 ± 0.002) and 2.92 ± 1.23% (*R*^2^ = 0.998 ± 0.002), respectively (Fig. 5a,b). Transverse models using the maximum normal strain damage criterion gave better fits overall (Fig. 5d), but were not significantly different than the von Mises stress damage criterion (Fig. 5c) (*p* = 0.07), with average NRMSEs of 9.89 ± 5.83% (*R*^2^ = 0.96 ± 0.05) and 13.40 ± 6.09% (*R*^2^ = 0.93 ± 0.06), respectively. No significant differences existed in ultimate stress or ultimate strain between the models and experiments, for either loading orientation (*p* > 0.99) (Table 2).

**﻿Table 2:** Comparison of mechanical behavior between damage models and experiments (sample size for each cell = 20). All strain values are reported as Lagrange strain.

	Longitudinal			Transverse		
	Experiment	Model (von Mises stress)	Model (max normal strain)	Experiment	Model (von Mises stress)	Model (max normal strain)
Ultimate tensile force (N)	2.25 ± 1.42	2.25 ± 1.41	2.23 ± 1.39	0.100 ± 0.066	0.103 ± 0.068	0.100 ± 0.066
Ultimate tensile stretch (grip-to-grip)	1.155 ± 0.052	1.153 ± 0.053	1.155 ± 0.052	1.493 ± 0.214	1.491 ± 0.212	1.491 ± 0.216
Normal strain *E*_*yy*_ (tear region at UTS)	0.29 ± 0.14	0.26 ± 0.11	0.27 ± 0.10	3.64 ± 3.30	1.00 ± 0.46*	1.02 ± 0.54*
Normal strain *E* (tear region at UTS)	0.01 ± 0.16	− 0.09 ± 0.07*	− 0.07 ± 0.03*	**− **0.21 ± 0.31	**− **0.15 ± 0.06	**− **0.15 ± 0.06
Shear strain *E* (tear region at UTS)	0.20 ± 0.10	0.14 ± 0.12	0.09 ± 0.06*	0.529 ± 0.620	0.017 ± 0.011*	0.015 ± 0.008*
1st principle strain (tear region at UTS)	0.42 ± 0.14	0.31 ± 0.16*	0.29 ± 0.12*	3.78 ± 3.35	1.00 ± 0.46*	1.02 ± 0.54*
2nd principle strain (tear region at UTS)	**− **0.13 ± 0.10	**− **0.14 ± 0.12	**− **0.10 ± 0.05	**− **0.34 ± 0.08	− 0.15 ± 0.06*	− 0.15 ± 0.06*
Max shear strain (tear region at UTS)	0.28 ± 0.10	0.23 ± 0.13	0.19 ± 0.08*	2.06 ± 1.70	0.57 ± 0.25*	0.59 ± 0.29*

Bolded values with an ^*^ are significantly lower than experimental data within either the Longitudinal or Transverse group (*p* < 0.001).

### Tear region strain

**Figure 6:**
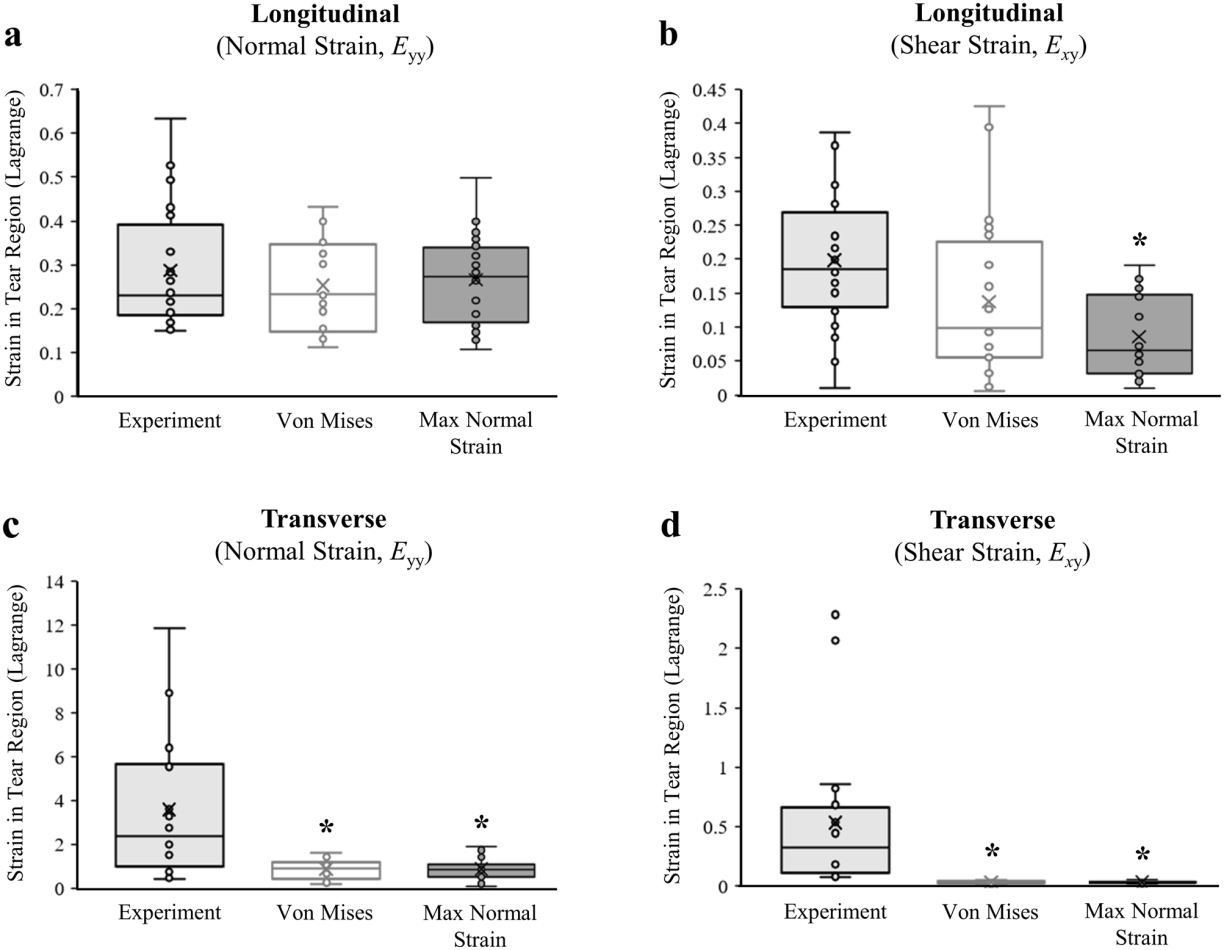
Comparison of strains in the tear region (ROI) between experiments measured using DIC and FE models generated with von Mises stress or maximum normal strain damage criterion. (**a**) Both damage criteria gave close predictions to the normal axial strains for the longitudinal loading condition, but (**b**) the maximum normal strain damage criterion significantly underpredicted the shear strains. (**c**) When loading transverse to the fiber direction, both models significantly underpredicted normal axial strains and (**d**) shear strains. *Significantly less than experimentally measured strains (*p* < 0.001). The ‘x’ in the box plot = average strain.

For the longitudinal group, normal axial strains (*E*_yy_) in the tear region were not significantly different than experimentally measured strains for either the von Mises stress or maximum normal strain damage criterion (*p* = 0.72, *p* = 0.87, respectively) (Fig. 6a, Table 2). The shear strains (*E*_xy_) predicted by the longitudinal models using the von Mises stress damage criterion were approximately 30% lower than experiments (*p* = 0.16), and were approximately 50% lower than experiments when using the maximum normal strain damage criterion (*p* = 0.003) (Fig. 6b, Table 2). For the transverse group, models using both damage criteria significantly underpredicted the experimental normal axial strains and shear strains (*p* < 0.001) (Fig. 6c,d; Table 2). The contraction of the specimens within the tear region (*E*_xx_) for all models was significantly less than experiments for the longitudinal group (*p* < 0.05), but not for the transverse group (*p* > 0.50).

**Figure 7:**
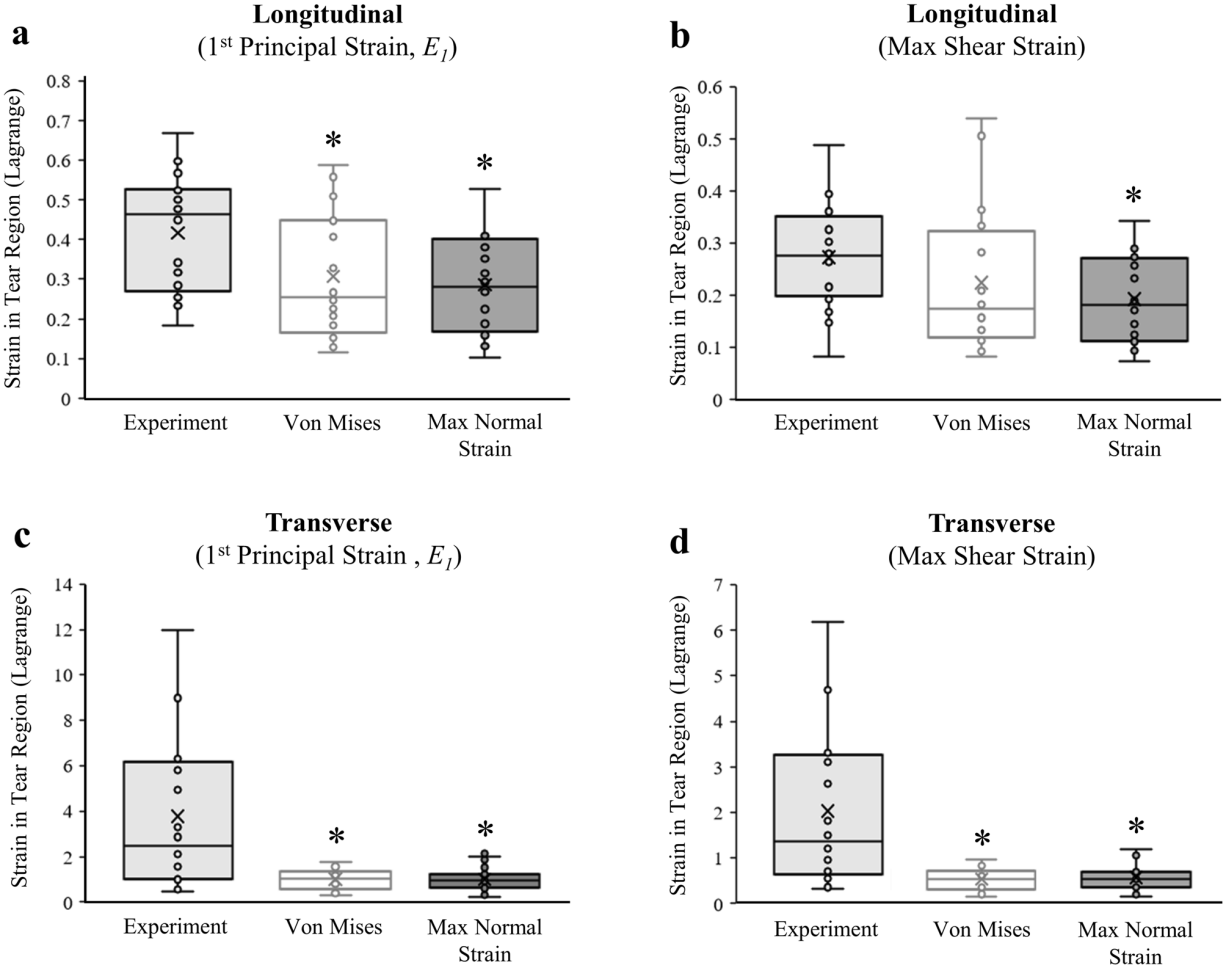
Comparison of principal strains in the tear region (ROI) between experiments measured using DIC and FE models generated with von Mises stress or maximum normal strain damage criterion. (**a**) Both damage criteria significantly under predicted the first principal strains for the longitudinal loading condition, but (**b**) only the maximum normal strain damage criterion significantly underpredicted the max shear strains. (**c**) When loading transverse to the fiber direction, both models significantly underpredicted first principal strains and (**d**) max shear strains. *Significantly less than experimentally measured strains (*p* < 0.05). The ‘x’ in the box plot = average strain.

For longitudinal tests, first principal strains predicted by both the von Mises stress damage criterion and the maximum normal strain damage criterion were significantly lower than the experimental strains (*p* = 0.042,* p* = 0.013, respectively) (Fig. 7a). The maximum shear strains predicted by both damage criteria were also less than experimental strains, but only maximum normal strain damage criterion were significantly less (*p* = 0.046) (Fig. 7b). The second principal strains predicted by both damage criteria were similar to experimental strains (*p* > 0.5; Table 2). For transverse tests, first and second principal strains, as well as max shear strains predicted by both damage criteria were significantly lower than experimental strains (*p* < 0.001) (Fig. 7c,d; Table 2).

For transverse models, both damage criterion predicted tears to propagate straight across the coupon at a 0° angle (i.e. perpendicular to the loading axis). For longitudinal models, the maximum normal strain damage criterion similarly predicted a 0° tear angle, however, longitudinal models using the von Mises stress damage criterion had tears initiate near the fillet and propagate at approximately 70°, measured perpendicular to the loading axis (an angle of 90° would be parallel to the loading axis), before changing angle at the midline of the coupon, where it continued propagating to the opposite boundary at 0 degrees (Fig. 4b). The average damage in these tear regions at UTS for longitudinal models was 0.26 ± 0.10 and 0.28 ± 0.16 for the von Mises stress and maximum normal strain damage criteria, respectively. For transverse models, the average damage was 0.59 ± 0.17 for both damage criteria.

## Discussion

The objective of this study was to determine if finite element models using hyperelastic damage constitutive equations could simulate tears in the fibers and ground substance of human meniscus. We found that von Mises stress and maximum normal strain damage criteria were able to simulate the experimental anisotropic stress–strain behavior (Fig. 5), but in general, both damage criteria underpredicted the strains in the tear region. The one exception was that both damage criteria were able to reasonably predict normal strains along the loading axis for longitudinal tests. Also, the von Mises stress damage criterion was uniquely able to approximate the distinct tear angles for both fiber and ground substance failures. Overall, our findings partially supported our hypothesis and demonstrate limitations in using continuum level damage mechanics to simulate failure in soft fibrous tissue.

The excellent fits of our models to experimental force–displacement curves verified that the selected constitutive formulations were appropriate for modeling the grip-to-grip mechanical failure behavior of human meniscus. When loading the fibers (longitudinal tests), the piecewise strain energy function (Eq. 3) was able to fit the non-linear toe region^42^ and linear region of the experimental data, and the damage formulation was able to fit the experimental stress-softening region where collagen damage accumulates^43^. As a result, our longitudinal models had an average *R*^2^ value (> 0.99) equal or better than other CDM modeling papers we surveyed for soft tissue^18,19,44^. When loading the ground substance (transverse tests), our model fits to experiments had slightly lower average *R*^2^ values (> 0.93). Interestingly, many of the transverse experiments displayed localized stress peaks prior to UTS (Fig. 5c,d), possibly indicating the sporadic failure of tie fiber groups, which run normal to the circumferential direction^8^, and are stiffer and less extensible then the ground substance. We were able to recreate these localized peaks in our FE model by setting an upper limit to ground substance damage via *D*_*max*_ (Eq. 4). By limiting the maximum amount of damage, elements subjected to high strain concentrations that experience rapid damage would stabilize once maximum damage was reached, and would eventually support greater loads as the tissue continued to stretch. The ability of our model to match this experimental behavior supports the use of CDM for modeling ground substance failure, rather than fracture mechanics. This conclusion supports work by Peloquin et al., who conducted experiments with cracked meniscus tissue and concluded that fracture mechanics was not an appropriate failure analysis method for meniscal tissue^45^.

The experimental validation of local strains in the longitudinal models exposed strengths and weaknesses of using CDM to model fiber failures. We were initially encouraged that both damage criteria could fairly accurately predict normal strains along the loading axis for longitudinal tests (Fig. 6a), but when we examined the principal strains (Fig. 7a), both damage criteria underpredicted the 1st principal strain in the tear region by approximately 30%. For comparison, previous studies that used DIC to validate an FE model of relatively hard materials (e.g. bone, thermoplastics) reported errors ranging from 10 to 24%^22,23,28^, therefore our error is larger than desired. Our error is partially explained by the FE model underpredicting the *E*_xy_ shear strains that occur on the surface normal to the x-axis (Fig. 6b). The larger *E*_xy_ shear strains in the experiments likely develop from collagen fiber sliding^46^ that could potentially be simulated with a micromechanics model. Since our model underpredicted 1st principal strain, we would have expected the 2nd principal strain to similarly be underpredicted, but on the contrary, the model predicted 2nd principal strains were a good match to experiments (Table 2). This disparity between principal strains indicates that our longitudinal models overpredicted the amount of lateral contraction during axial elongation, and that incompressibility is a poor physiological assumption when modeling the necking behavior that occurs in the tear region of fiber failures.

The experimental validation of strains in the transverse models demonstrated that a hyperelastic damage formulation is unable to capture the considerable extensibility of the ground substance in the tear region. In experiments, the ground substance elongated by more than 2.5 times its original length at UTS, while in the models, the ground substance elongated by only approximately 1.75 times its original length at UTS (Table 2). This difference can be explained by discrepancies in the strain concentrations between the transverse experiments and models. The region of high strain concentration in the transverse experiments was a tight band of approximately 0.2 mm that propagated across the specimen surface, while the region of high concentration in the transverse models was a broad band of approximately 1.4 mm (Fig. 4b,c). Our mesh convergence study shows that further mesh refinement in the tear region would result in only a nominal improvement in predicted strains and would not resolve this limitation (Fig. 2a). Importantly, regularization methods commonly used to help FE damage models converge would likely only exacerbate this strain discrepancy, as these methods spread out localized deformation to prevent premature model termination^47,48^. A potential solution to simulate a tighter band of stress concentrations with CDM is to model region-dependent material parameters^49^ that effectively model localized defects within the tissue.

We evaluated two damage criteria in this study and found that von Mises stress offers two distinct advantages for modeling failure in soft tissue. First, longitudinal models that used von Mises stress damage criterion had slightly better overall predictions of 1st principal strain and maximum shear strain compared to models using maximum normal strain damage criterion (Fig. 7a,b, Table 2), although these differences were not significant. Second, models using von Mises stress damage criterion were better able to recreate the experimental tear angles. Longitudinal models with von Mises stress damage criterion had tears that initiated at the narrow section of the coupon fillet and propagated at a steep oblique angle until changing directions to propagate perpendicular to the loading axis (Fig. 4b). This tear pattern is indicative of a classic cup-and-cone failure pattern seen in ductile materials^31^, and was consistent with a subset of specimens in our previous experimental work that exhibited bimodal tear angles, characterized by steep initial tear angles that similarly “flattened” or changed direction near the coupon center axis^30^. The von Mises stress damage criterion was also able to model the “flat” tear angle of the transverse specimens near the coupon midsubstance where necking minimizes the cross-sectional area, thus maximizing 1st principal stress. Mathematically, since the 2nd and 3rd principal stresses are negligible near the midsubstance (simple tension), the von Mises equation simplifies to a maximum normal stress damage criterion and the tear would propagate perpendicular to the loading axis. It is therefore possible to use von Mises stress damage criteria to model the anisotropic tear patterns of meniscus, and other transversely isotropic soft fibrous tissues (e.g. ligament, tendon), however, different damage parameters would need to be used for the fibers and ground substance (Table 1). Not surprisingly, the maximum normal strain damage criterion simulated tears perpendicular to the loading axis near the midsubstance in both longitudinal and transverse groups (Fig. 4c), as this region experiences the most necking and axial strain. Maximum normal strain damage criterion would thereby be an appropriate model to predict tear patterns in the ground substance, but not the fibers.

This project was innovative by being the first to quantify whether a damage model can accurately predict the deformation in the tear region of soft tissue. Other soft tissue damage models have simulated the physiological deformation of the overall structure^18,19^, but not the localized deformation within the tear region. By comparing the model predicted surface strains and tear angles to the experimentally measured ones, we were able to determine whether the mathematical mechanisms driving model predicted failure are physiologically relevant. To our knowledge, this is also the first FE study of human meniscus to successfully simulate stress–strain curves to UTS (Fig. 5), as previous FE models of the whole meniscus disregarded any failure or softening behavior^11,50^. These novel contributions help advance scientific efforts to develop and validate computational tools that can reliably simulate mechanical failure in connective tissue, and thereby allow clinicians and researchers to visualize model outcomes with direct clinical significance (i.e. tissue tears) with a goal of designing new treatment and prevention strategies for meniscal injuries and other musculoskeletal disorders.

This work had notable limitations. First, we were unable to utilize FEBio’s parameter optimization plug-in to calibrate the damage parameters to the experimental force–displacement curves, as any combination of parameters that resulted in early model termination would halt the optimization routine. We instead manually fit these damage parameters to achieve a set of objective success criteria, and this resulted in force–displacement curves with excellent fits (Fig. 5, Table 2). Second, this study used a large number of linear tetrahedral elements and the use of other element types (e.g. quadratic tetrahedral, linear or quadratic hexahedral) may result in different strain calculations^51^. Next, our finite element models only investigated tears that develop under tensile loading, and did not consider the effects of compression on meniscus failure. The models also did not consider variable loading rates, which would require the implementation of a viscoelastic constitutive framework. Lastly, our model validation study used two-dimensional surface strains and may have missed important phenomena in out-of-plane strains and tear patterns. However, we did account for this limitation by only comparing the two-dimensional surface strains and tear patterns of our finite element model to the experimental results.

In conclusion, this study has quantified the ability of continuum damage FE models to predict tearing in meniscal tissue subjected to tensile loads. This work provides a benchmark for the ongoing development and validation of computational models that accurately simulate tears in soft fibrous tissues.

## Data Availability

All finite element models and experimental results used in the study are available upon request. Please contact the corresponding author (TJL).
